# Laparoscopic redo fundoplication for intrathoracic migration of wrap

**DOI:** 10.4103/0972-9941.37195

**Published:** 2007

**Authors:** G S Maheshkumar, Kalpech Jani, M V Madhankumar, C Palanivelu

**Affiliations:** Department of Minimal Access Surgery, GEM Hospital, 45A, Pankaja Mill Road, Coimbatore - 641 045, India

**Keywords:** Laparoscopic fundoplication, laparoscopic redo fundoplication, wrap migration

## Abstract

Laparoscopic fundoplication is fast emerging as the treatment of choice of gastro-esophageal reflux disease. However, a complication peculiar to laparoscopic surgery for this disease is the intrathoracic migration of the wrap. This article describes a case of a male patient who developed this particular complication after laparoscopic total fundoplication. Following a trauma, wrap migration occurred. The typical history and symptomatology is described. The classical Barium swallow picture is enclosed. Laparoscopic redo fundoplication was carried out. The difficulties encountered are described. Postoperative wrap migration can be suspected clinically by the presence of a precipitating event and typical symptomatology. Confirmation is by a Barium swallow. Treatment is by redo surgery.

## INTRODUCTION

Laparoscopic antireflux surgical procedures were introduced into clinical practice a little more than a decade ago. Today, they constitute a well-established treatment modality for gastro-esophageal reflux disease. Typical of many laparoscopic operations, antireflux procedures evolved with time and underwent several technical refinements. There continues to be a considerable debate on some of the technical aspects of these procedures and on the long-term difference in outcome between partial and complete fundoplication. Failures of antireflux procedures occur in 5% to 10% of the patients.[1] Postoperative intrathoracic wrap migration is the most frequent morphological complication after laparoscopic antireflux surgery.[1] Re-do laparoscopic antireflux operations are technically challenging but feasible in experienced hands.[2] We report our experience with laparoscopic management of a failed fundoplication.

## CASE REPORT

**Figure 1:**
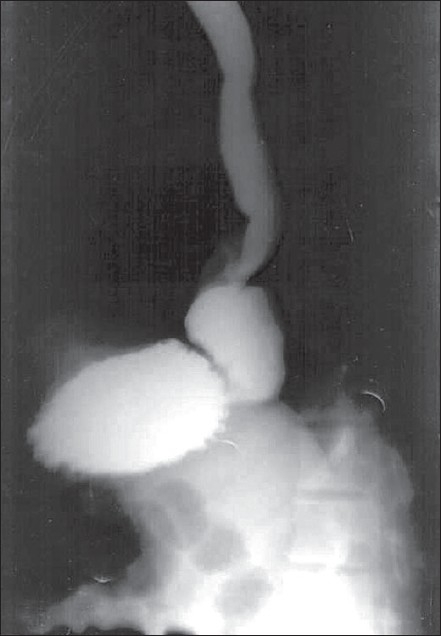
Barium meal picture showing intrathoracic migration of fundoplication wrap

**Figure 2:**
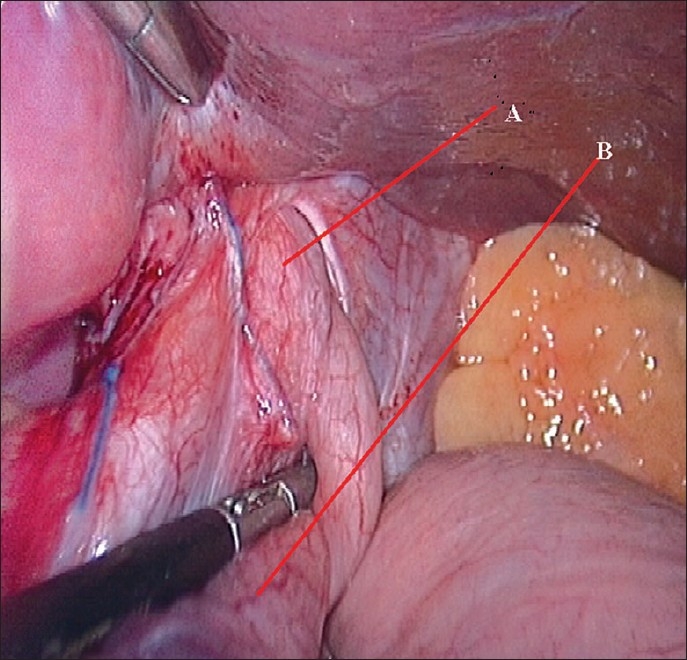
A. Esophagus. B: The herniated wrap

**Figure 3:**
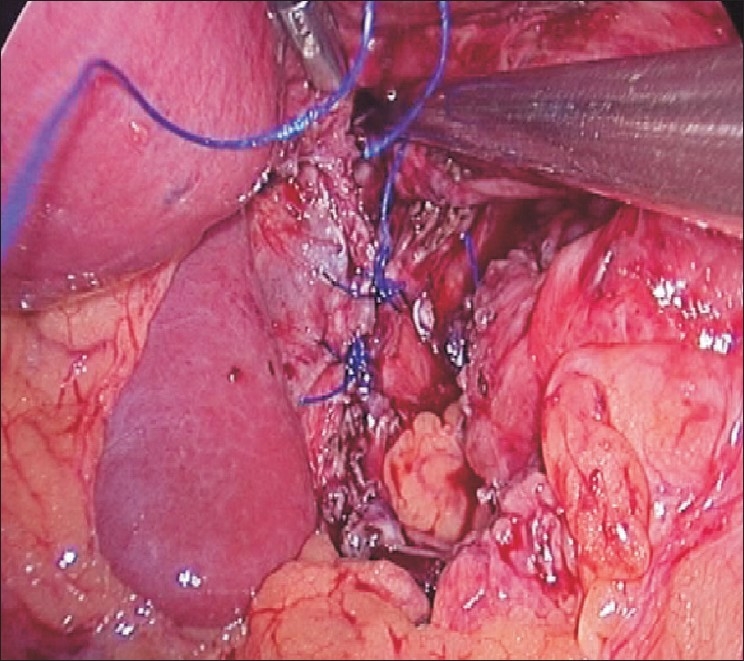
Completed re-do hiatal repair

A 38-year-old male patient presented with pain and retrosternal discomfort following each meal. Three months previously, he had undergone a laparoscopic Nissen fundoplication with crurorhaphy for a sliding hiatus hernia with gastro-esophageal reflux, diagnosed on endoscopy, manometry and pH study. His postoperative period had been unremarkable and he had been discharged on the second postoperative day. He had remained symptom-free for two months with periodic follow-up (once in 15 days). A month ago, he had a fall on the stairs and the banister of the staircase had struck him in the upper abdomen. He had consulted a local surgeon, who kept him under observation for 48h and then discharged him. Thereafter, he noticed severe pain and epigastric discomfort that increased after meals. At our institute, he was investigated again. Endoscopy revealed a gastric pouch above the hiatal constriction. Barium study confirmed the diagnosis of intrathoracic migration of the fundoplication. [Figure 1]. He was taken for laparoscopic surgery under general anesthesia using the same port sites. Intraoperatively, the crural repair was found to have disrupted, with migration of the wrap into the thorax [Figure 2]. The wrap was reduced back into the abdomen. Some adhesions had to be divided with ultrasonic shears. The crura were re-approximated posteriorly with 1-0 polypropylene interrupted sutures [Figure 3] and the wrap was fixed to the crura in a similar fashion. Intracorporeal suturing technique was used. Postoperatively, the patient had a smooth course, taking liquids on the next day and was discharged after 48h. After three months of follow-up, he is free from symptoms and is able to take a normal diet.

## DISCUSSION

In spite of the growing popularity of laparoscopic fundoplication, this procedure is not without its complications.[1,2] Intrathoracic wrap migration is one of the most frequent reported adverse event.[2]

Most patients presenting with a failed fundoplication complain of recurrent heartburn, dyphagia or both.[3] Less frequent complaints were persistent nausea and vomiting, diarrhea, severe gas bloat symptoms and shoulder or chest pain. Not all patients with this condition have symptoms. Asymptomatic patients may be managed expectantly.[3] However, acute complication like volvulus of the incarcerated stomach can occur and the need for emergency surgery must be explained to the patient.[4] Intrathoracic wrap migration in the immediate postoperative period can occur due to failure or inadequate crural closure, insufficient esophageal mobilization, shortened esophagus or severe retching soon after surgery.[2,5] Weakness of the crural closure, upwards tension on the esophagus and a sudden increase in intraabdominal pressure due to a fall or blow to the upper abdomen can lead to late wrap migration. A recent study has shown that placement of esophagocrural sutures and minimization of the dissection around the esophagus results in a more than two-fold reduction in the risk of wrap transmigration after laparoscopic Nissen fundoplication.[6]

It is important to determine either preoperatively or during re-exploration why the fundoplication migrated up into the chest. If a shortened esophagus is a possibility, both the patient and the surgical team should be prepared to proceed with an esophageal lengthening procedure. This may require conversion to an open laparotomy or thoracotomy.

Though corrective surgery has been performed safely through the abdomen,[2] some authorities believe that a transthoracic approach is more appropriate due to the presence of fewer adhesions and safer access to the esophagus.[7]

Tension-free hiatoplasty using polypropylene mesh has been recommended in all cases to prevent this complication.[7] But even this modification is not without its side-effects and at least one report of intra-esophageal mesh migration has been reported.[8] An alternative is the use of biodegradable mesh for crural re-enforcement. A prospective comparative study has suggested that hiatal hernia repair reinforced with an acellular dermal matrix patch may reduce the incidence of recurrent herniation and wrap migration.[9] In addition, the increase in postoperative dysphagia, chest pain and esophageal erosions associated with non-degradable mesh has not been observed in those with an acellular dermal matrix patch. The most reasonable way to avoid intra-thoracic wrap migration is performance by an experienced laparoscopic surgeon, adequate esophageal mobilization, secure diaphragmatic closure, esophageal lengthening (applied selectively) and avoidance of events leading to increased intraabdominal pressure.[2]

## CONCLUSION

Intrathoracic wrap migration following laparoscopic fundoplication is the most common morphological complication of the surgery. It can be suspected clinically by the history of a precipitating event and the typical symptomatology. Redo fundoplication by the laparoscopic approach is feasible, though difficult and is safe in expert hands.
